# Natural Indoles, Indole-3-Carbinol (I3C) and 3,3’-Diindolylmethane (DIM), Attenuate Staphylococcal Enterotoxin B-Mediated Liver Injury by Downregulating miR-31 Expression and Promoting Caspase-2-Mediated Apoptosis

**DOI:** 10.1371/journal.pone.0118506

**Published:** 2015-02-23

**Authors:** Philip B. Busbee, Mitzi Nagarkatti, Prakash S. Nagarkatti

**Affiliations:** 1 Department of Pathology, Microbiology and Immunology, University of South Carolina School of Medicine, Columbia, South Carolina, United States of America; 2 WJB Dorn Veterans Affairs Medical Center, Columbia, South Carolina, United States of America; Faculty of Medicine & Health Sciences, UNITED ARAB EMIRATES

## Abstract

Staphylococcal enterotoxin B (SEB) is a potent superantigen capable of inducing inflammation characterized by robust immune cell activation and proinflammatory cytokine release. Exposure to SEB can result in food poisoning as well as fatal conditions such as toxic shock syndrome. In the current study, we investigated the effect of natural indoles including indole-3-carbinol (I3C) and 3,3’-diindolylmethane (DIM) on SEB-mediated liver injury. Injection of SEB into D-galactosamine-sensitized female C57BL/6 mice resulted in liver injury as indicated by an increase in enzyme aspartate transaminase (AST) levels, induction of inflammatory cytokines, and massive infiltration of immune cells into the liver. Administration of I3C and DIM (40mg/kg), by intraperitonal injection, attenuated SEB-induced acute liver injury, as evidenced by decrease in AST levels, inflammatory cytokines and cellular infiltration in the liver. I3C and DIM triggered apoptosis in SEB-activated T cells primarily through activation of the intrinsic mitochondrial pathway. In addition, inhibitor studies involving caspases revealed that I3C and DIM-mediated apoptosis in these activated cells was dependent on caspase-2 but independent of caspase-8, 9 and 3. In addition, I3C and DIM caused a decrease in Bcl-2 expression. Both compounds also down-regulated miR-31, which directly targets caspase-2 and influences apoptosis in SEB-activated cells. Our data demonstrate for the first time that indoles can effectively suppress acute hepatic inflammation caused by SEB and that this may be mediated by decreased expression of miR-31 and consequent caspase-2-dependent apoptosis in T cells.

## Introduction

Staphylococcal enterotoxin B (SEB) is a toxic superantigen (SAG) and major virulence factor secreted by the bacterium *Staphylococcus aureus* (*S*. *aureus*), which causes nosocomial infections and community acquired diseases [1–2]. Exposure of humans to SEB, which can be inhaled or ingested, can result in severe food poisoning and even fatal conditions, such as toxic shock syndrome [3]. SEB is extremely stable in acidic environments, such as the gastrointestinal tract, and is highly resistant to heat and proteolytic digestion [4]. These properties, along with the fact that SEB is easily aerosolized, resulted in the Centers for Disease Control (CDC) classifying this superantigen as a category B priority agent for potential use as a biological weapon [5].

SEB is classified as aSAG because it bypasses normal processing by antigen-presenting cells (APCs), but instead interacts outside of the peptide-binding groove of major histocompatibility complex class II (MHC II) molecules with certain external Vβ domains located on T cell receptors (TCR). This results in rapid T cell activation without regard to antigen specificity [6–7]. While normal antigens are estimated to activate approximately 0.1% of host T cells, superantigens can activate up to 30% [8]. Such massive T cell activation leads to uncontrolled proinflammatory cytokine release, termed as a cytokine storm, including the release tumor necrosis factor-alpha (TNF-α), interferon-gamma (IFN-γ), and interleukins (IL)-1,2,6,8, and 12 [9–11]. Despite improvements in healthcare, *S*. *aureus* exposure still results in 20–30% mortality in the developed world, partly because of the ability of this bacterium to acquire antibiotic resistance determinants [12]. Therefore, a more effective treatment would involve controlling the rapid T cell activation and cytokine storm by the virulent factors produced by *S*. *aureus*, such as SEB.

Indole-3-carbinol (I3C), formed from the enzymatic breakdown of glucosinolate glucobrassicin by the enzyme myrosinase, is a natural indole compound found in cruciferous vegetables. 3,3’-diindolylmethane (DIM), is the major byproduct produced when I3C undergoes self-condensation reactions in acidic environments, such as the gut [13]. Chemical structures of these natural indole compounds can be found in S1 Fig. These natural compounds have been shown to exert a variety of beneficial effects which include anti-cancer [14], anti-microbial [15], immunomodulatory [16–17], and anti-inflammatory properties [18–20]. In our previous studies, we found that I3C and DIM were able to reduce T cell activation and proinflammatory cytokine release after exposure of C57BL/6 mice to SEB administered into the footpad [21]. In addition, I3C and DIM caused a decrease in HDAC-I but not HDAC-II in SEB-activated T cells, thereby suggesting that indoles may inhibit SEB-mediated T cell activation by acting as HDAC-I inhibitors [21]. We have also shown that indoles can effectively suppress neuroinflammation seen in mice with experimental autoimmune encephalomyelitis by inducing Tregs while suppressing Th17 cells [20].

Despite such studies, whether indoles can protect an organ such as the liver from acute and robust inflammation induced by SEB has not been investigated previously. In the current study, we demonstrate that indoles can attenuate liver inflammation and injury caused by SEB through activation of a unique pathway of apoptosis involving the induction of caspase-2. We also show that indoles can suppress SEB-mediated T cell activation and cytokine production independent of caspase-2 pathway. Lastly, we were able to identify microRNA (miR), small single-stranded noncoding molecules capable of suppression of complementary mRNA targets [22], which affect caspase-2. Specifically, we found that miR-31 directly targets caspase-2. Together these studies further attest that plant-derived indoles are potent anti-inflammatory agents.

## Materials and Methods

### Animals

Female adult C57Bl/6 mice were purchased from National Cancer Institute, National Institute of Health [Frederick, MD] and bred under appropriate conditions. All protocols were approved by the University of South Carolina Institutional Animal Care and Use Committee [IACUC] and mice were housed under specific pathogen-free conditions.

### Effects of I3C and DIM on SEB-induced acute liver injury mice

To test the efficacy of treatment with I3C and DIM in an *in vivo* SEB-induced acute liver injury mouse model, SEB, purchased from Toxin Technologies (Sarasota, FL), was injected intraperitonally (i.p.) into age- and weight-matched female C57BL/6 mice at a dose of 40µg in PBS, as described previously [23]. Inasmuch as mice are more resistant than humans to bacterial toxins [9], the mice used in these experiments were first sensitized by giving them an i.p. injection of 20mg of D-galactosamine (Dgal) in PBS 30 minutes prior to SEB injection, as described [23]. For treatment groups, I3C and DIM, purchased from Sigma-Aldrich (St. Louis, MO), were administered i.p. at 40mg/kg, a dose established in our previous studies [21], in a total volume of 100µl in appropriate vehicle (2% DMSO in corn oil). I3C and DIM were given once 24 hours prior to SEB injection and the second dose the following day 1 hour prior to injection of Dgal and SEB. Mice were monitored daily and euthanized by overdose of isoflurane followed by cervical dislocation prior to blood and tissue collection, as approved by the University School of Medicine IACUC. Mice were daily watched for any signs of distress and any moribund mice were immediately euthanize. Liver infiltrating mononuclear cells were isolated and counted 24 hours after SEB challenge by Percoll density separation as previously described [23]. Blood was collected at 8 and 24 hours and sera were separated and stored at -20°C. Liver enzyme aspartate transaminase (AST) levels were measured at 340nm by spectrophotometric method from sera collected at 8 hours using a commercially available AST assay kit from Pointe Scientific (Canton, MI) as described previously [23] to assess liver damage. Liver histology was obtained by harvesting livers 24 hours after SEB injection and fixing them in 10% formalin. Fixed tissues were embedded in paraffin, cut into 5µm sections, deparaffinzied in xylene, serially diluted in decreasing concentrations of ethanol, and stained with hematoxylin-eosin (H&E) for examination under a light microscope for cell infiltration. Experimental groups consisted of five mice each, and each study was repeated at least three times.

### Effects of I3C and DIM on splenocytes *in vitro*

Spleens were excised from C57BL/6 mice and placed in complete RPMI 1640 media supplemented with heat inactivated 10% fetal bovine serum, 10mM L-glutamine, 10mM HEPES, 50µM β-mercaptoethanol, and 100µg/ml penicillin/streptomycin. Tissues were homogenized into single-cell suspensions and subjected to red blood cell lysis. Cells were plated in a 96-well plate in complete media at 1x10^6^ cells per well for 24 hours at 37°C and 5% CO_2_ with or without SEB-stimulation (1µg/ml) and with vehicle, I3C or DIM (100µM). Vehicle for all compounds was dimethyl sulfoxide (DMSO), with a total volume of never exceeding 0.5% DMSO in complete medium per well. Cells were harvested and counted after 24 hours. To assess activation, cells collected from *in vitro* cultures were stained with CD69 antibody purchased from Biolegend (San Diego, CA) for flow cytometry analysis.

### Measurement of cytokines in culture supernatants and sera

Cell culture supernatants were collected after 24 hours from *in vitro* experiments as described above. For *in vivo* serum cytokine levels, serum was collected 24 hours after mice were injected with SEB or vehicle as described above. To measure cytokines from cells infiltrating the liver, mononuclear cells were isolated as described above. These cells were then plated in 96-well plates (1x10^6^ cells per well) for 24 hours in complete RPMI 1640 media (total volume of 200 µL) supplemented with heat inactivated 10% fetal bovine serum, 10mM L-glutamine, 10mM HEPES, 50µM β-mercaptoethanol, and 100µg/ml penicillin/streptomycin. Supernatants were collected from these *ex vivo* cultured cells after 24 hours for cytokine analysis. Cytokines levels were analyzed and quantified using individual enzyme-linked immunosorbent assay (ELISA) kits for IFN-γ, TNF-α, IL-2, and IL-6 purchased from Biolegend (San Diego, CA).

### Evaluation of apoptosis from *in vitro* cultures

Apoptosis was measured from *in vitro* cultures described above by terminal deoxynucleotidyl transferase dUTP nick end labeling (TUNEL), annexin-propidium iodide (PI), and 3,3′-dihexyloxacarbocyanine iodide (DiOC6) methods [24]. TUNEL was measured using a Fluorescein *In Situ* Cell Death Detection Kit from Roche (Indianapolis, IN) as per manufacturer’s instructions. Annexin-PI staining was performed by using a FITC Annexin V kit purchased from Biolegend (San Diego, CA) as per manufacturer’s instructions. DiOC6 was purchased from Molecular Probes (Eugene, OR), and cells were stained with 40nM of DiOC6 for 30 minutes at 37°C before analysis. For studies using caspase inhibitors, inhibitors for caspases-2, 3, 8, and 9 were purchased from Calbiochem (San Diego, CA) and incubated simultaneously with the various treatments described above at a concentration of 100µM. Cells from all these assays were analyzed using flow cytometry to detect apoptosis.

### RT-PCR for analysis

Expression of mRNA for caspase-2, Bcl-2, and miR-31 from 12-hour *in vitro* cultures and 24-hour *in vivo* isolated mononuclear cells was determined by quantitative real-time PCR (qRT-PCR). Samples from *in vitro* and *in vivo* experiments were collected as described above. mRNA was isolated using RNeasy kit from Qiagen (Valencia, CA), and cDNA was synthesized using miScript cDNA synthesis kit from Bio-Rad (Hercules, CA). For mRNA expression, qRT-PCR was carried out using SsoAdvanced SYBR green supermix from Bio-Rad (Hercules, CA) with mouse primers for caspase-2 (forward 5’-GCAACTCTACCTGTTCCCAG-3’ and reverse 5’-GAGAAGTCTCCATTGTGCCC-3’) and Bcl-2 (forward 5’-AGATGCGCAGGTTGGGGTGTG-3’ and reverse 5’-CTGCGTCCTCTGGTGGAGCCT-3’). Expression levels were normalized to β-actin (forward 5’-ATCATTGCTCCTGAGCG-3’ and reverse 5’-CAGCTCAGTAACAGTCCGCC-3’) mRNA levels. For validation of miRNA expression, quantitative RT-PCR was performed using miScript SYBR green PCR kit and mouse primers for miR-31 (MS00001407) and Snord96a (MS00033733) from Qiagen (Valencia, CA). Expression levels were normalized to Snord96a levels. Fold changes were calculated using the 2^−ΔΔCT^ method.

### Western blots for caspase-2 and Bcl-2

Whole cell lysates were prepared from 12-hour *in vitro* cell cultures and 24-hour *in vivo* isolated mononuclear cells using RIPA Lysis Buffer System purchased from Santa Cruz Biotechnology, Inc. (Santa Cruz, CA). Protein concentration was determined using Pierce BCA Protein Assay kit purchased from Thermo Scientific (Rockford, IL). Protein samples (10–15µg) were separated by SDS-page and transferred to nitrocellulose membranes using a semi-dry apparatus. Membranes were then placed in 5% dry milk blocking buffer for 1 hour at room temperature on a shaker. Membranes were then washed and incubated overnight at 4°C in primary antibodies for caspase-2 (1:200 dilution) and Bcl-2 (1:500 dilution). Primary antibody for caspase-2 was purchased from Enzo Life Sciences (Farmingdale, NY), and primary antibody for Bcl-2 was purchased from Santa Cruz Biotechnology, Inc. (Santa Cruz, CA). After the overnight incubation in primary antibodies, membranes were washed and incubated with secondary antibody (anti-mouse IgG) for 1 hour at room temperature. Membranes were then washed and incubated in developing solution (Pierce ECL Western Blotting Substrate) purchased from Thermo Scientific (Rockford, IL) for 1 minute. Western blots were quantified using ImageJ software, and relative expression of cleaved p19 caspase-2 and Bcl-2 was corrected against β-actin as a loading control.

### miRNA expression profiling and miRNA target analysis

Total RNA was isolated from 12-hour *in vitro* cultures activated with SEB (1µg/ml) and treated with or without I3C or DIM (100µM) as described above. The concentration and purity of the isolated RNA were determined using a spectrophotometer, and the integrity of the RNA was verified by Agilent 2100 BioAnalyzer (Agilent Tech, Palo Alto, CA). Profiling of miRNA expression from samples was performed using the Affymetrix GeneChip miRNA 3.0 array platform (Affymetrix, Santa Clara, CA). This array, composed of 2023 miRNA mouse probes and using the FlashTag biotin HSR hybridization technique, was performed as previously described [25]. A heatmap was generated by taking the log transformation of fluorescent intensities obtained from the hybridization. Ward’s method was used to carry out hierarchical clustering and similarities were measured using half square Euclidean distance. miRNA fold changes were obtained from the array and miRNAs with only a greater than 1.5-fold change were considered for further analysis. Predicted miRNA targets, alignments, and mirSVR scores were determined by using an online miRNA and miRNA database (microrna.org). Raw data obtained from the Affymetrix GeneChip miRNA 3.0 array platform was deposited in the ArrayExpress database (www.ebi.ac.uk/arrayexpress) under accession number E-MTAB-3185.

### Transfection with miR-31 mimic and inhibitor

Spleens were excised from C57BL/6 mice and cultured in complete RPMI 1640 media supplemented with heat inactivated 10% fetal bovine serum, 10mM L-glutamine, 10mM HEPES, 50µM β-mercaptoethanol, and 100µg/ml penicillin/streptomycin. Cells were seeded at 2 x 10^5^ cells per well in a 24-well plate and activated with SEB (1µg/ml). Cells were then transfected with either 20nM of synthetic mmu-miR-31–5p mimic (AGGCAAGAUGCUGGCAUAGCUG) or anti-mmu-miR-31–5p (AGGCAAGAUGCUGGCAUAGCUG) using HiPerfect Transfection Reagent from Qiagen (Valencia, CA) for 24 hours. Expression levels of caspase-2 and miR-31 were determined using qRT-PCR as described above. TUNEL and flow cytometry were used to study apoptosis in cell cultures transfected with either synthetic mmu-miR-31–5p mimic or anti-mmu-miR-31–5p.

### Statistical Analysis

GraphPad Prism software (San Diego, CA) was used for all statistical analysis. For the *in vivo* mouse experiments, 5 mice were used per experimental group. For *in vitro* assays, all experiments were performed in triplicate. For statistical differences, one-way ANOVA was calculated for each experiment, and Tukey’s post-hoc test was performed to analyze differences between groups, unless otherwise indicated. A *p* value of ≤ 0.05 was used to determine statistical significance.

## Results

### I3C and DIM attenuate SEB-induced acute liver injury

**Fig 1:**
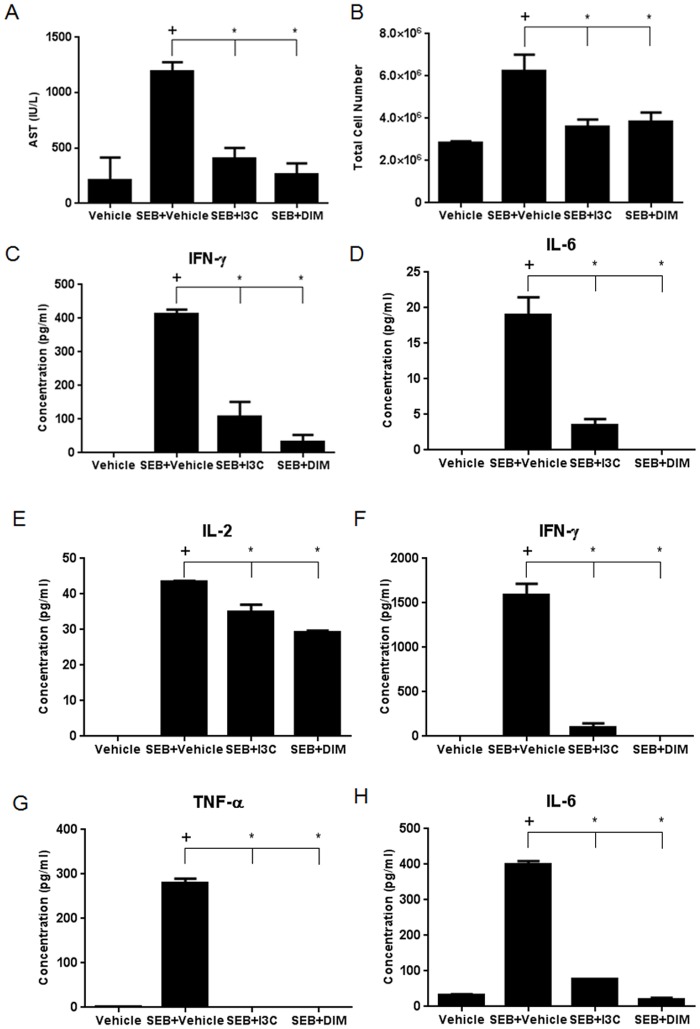
Treatment with I3C or DIM reduces AST levels, number of liver mononuclear cells, and proinflammatory cytokines in SEB-induced acute liver injury. Acute liver injury was induced in female C57BL/6 mice by injecting 20mg of Dgal i.p. followed 30 minutes later with an i.p. injection of 40µg of SEB (n = 5). Treatment groups were given an i.p. injection of I3C or DIM (40mg/kg) 24 hours and 1 hour prior to SEB injection. Serum was collected at 8 and 24 hours. AST levels at 8 hours were determined for evidence of liver damage (A). Mononuclear cells were isolated from mice after 24 hours and total cell number was calculated (B). Cytokine levels from serum isolated at 24 hours were detected by ELISAs for IFN-γ, IL-6, and IL-2 (C-E). Cytokine levels from supernatants of isolated liver mononuclear cells cultured for 24 hours were detected by ELISAs for IFN-γ, TNF-α, and IL-6 (F-H). Statistical significance (p-value <0.05) was determined using GraphPad Prism analysis software with one-way ANOVA and Tukey’s multiple comparison test (+ indicates significance compared to Vehicle group, and * indicates significance compared to SEB+Vehicle).

To study the effect of I3C and DIM on SEB-induced acute liver injury, we sensitized five mice per experimental with Dgal followed by i.p. injection of SEB, as described previously [23]. Additionally, these mice received I3C, DIM (40mg/kg), or vehicle 24 hours before and 1 hour prior to SEB challenge. As expected, injection of SEB into Dgal-sensitized mice resulted in an increase in AST levels in sera collected at 8 hours, an indication of acute liver injury. However, mice treated with I3C or DIM showed a significant decrease in the levels of AST (Fig. 1A). Next, we evaluated the number of mononuclear cells isolated from the livers of these mice to determine whether or not I3C or DIM could reduce the number of infiltrating cells induced by SEB challenge. SEB caused an increase in the number of infiltrating mononuclear cells in the liver compared to vehicle-treated mice, while both I3C and DIM were able to reduce this number substantially (Fig. 1B).

SEB, by nature of its superantigenicity, triggers the production of proinflammatory cytokines such as IFN-γ, TNF-α, IL-2, and IL-6 [26–27]. To that end, we analyzed serum cytokine levels at 24-hours following SEB challenge. While TNF-α was not detected in the serum in any of the groups at this time point, we did note an increase in IFN-γ, IL-2, and IL-6 in mice challenged with SEB when compared to vehicle-treated. In contrast, treatment with either I3C or DIM resulted in significant reduction in the detected circulating cytokines (Fig. 1C-E). To test cytokine production occurring in the liver microenvironment, we isolated mononuclear cells from this organ and cultured them for 24 hours. The supernatants were then analyzed for the production of the aforementioned cytokines. These data demonstrated that mice injected with SEB had significant increases in IFN-γ, TNF-α, and IL-6 produced by liver mononuclear cells when compared to vehicle, whereas treatment with I3C or DIM caused marked reduction in these cytokines (Fig. 1F-H).

**Fig 2:**
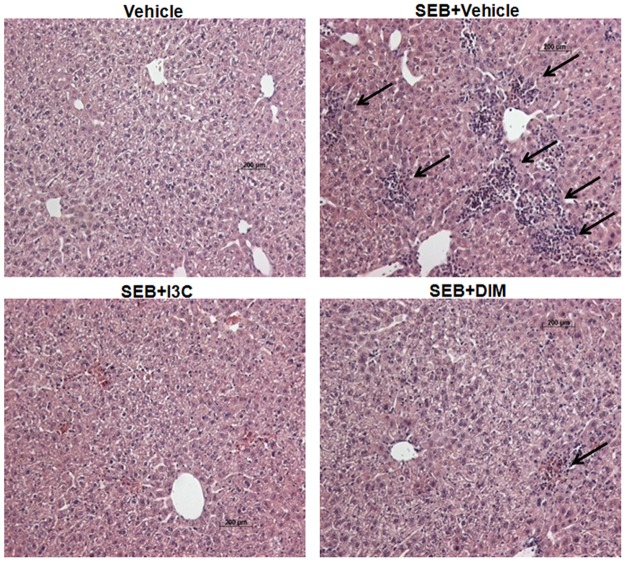
Treatment with I3C or DIM reduces cellular infiltration in livers of mice exposed to SEB. SEB-induced acute liver injury was induced in female C57BL/6 mice and these mice were treated with I3C and DIM as described in Fig. 1 legend. Liver sections isolated from mice 24 hours after SEB treatment were stained with H&E to detect cellular infiltration. Black arrows highlight areas with evidence of cellular infiltration.

Histological examination of formalin-fixed liver tissues was also performed. Mice sensitized with Dgal and treated with only vehicle had normal tissue morphology and showed no signs of cellular infiltration. Liver tissue samples from mice challenged with SEB however showed significant amounts of infiltrating cells, which was greatly reduced and almost completely ablated in livers of mice treated with I3C or DIM (Fig. 2). Collectively, these data showed that I3C and DIM were very effective at attenuating SEB-induced acute liver injury, specifically by reducing the number of cells infiltrating into the liver and reducing the levels of proinflammatory cytokines.

### I3C and DIM induce apoptosis via the mitochondrial pathway in cells activated with SEB

**Fig 3:**
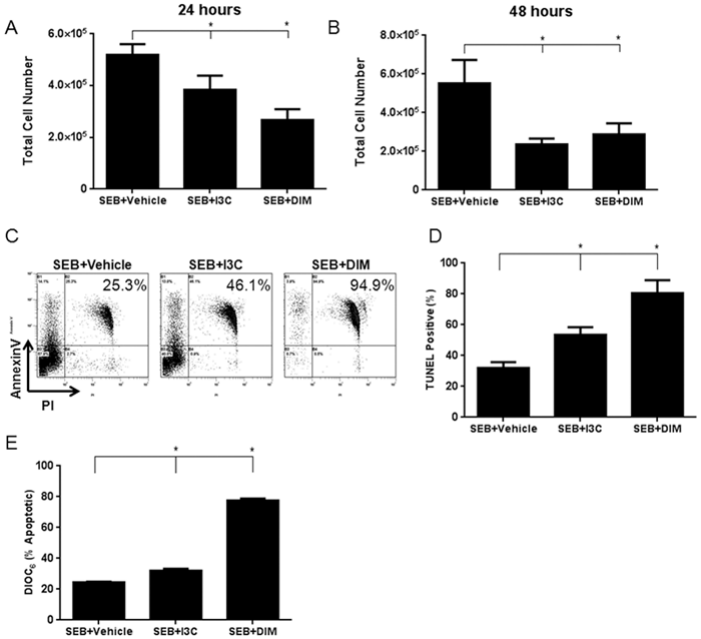
Treatment with I3C or DIM induces apoptosis in SEB-activated cells. Splenocytes from C57BL/6 mice were activated with SEB (1μg/ml) in vitro in 96-well plates in complete culture media in the presence or absence of I3C or DIM (100μM). Cells were collected and counted to determine cell number (A-B). Representative plots of 24 hour cultures stained with Annexin V/PI are shown (C). In addition, cells were stained for TUNEL after 24 hour culture and analyzed by flow cytometry to detect apoptosis (D). The cells were also stained with DiOC6 to determine if the observed apoptosis was mediated through the mitochondrial pathway (E). Statistical significance (p-value <0.05) was determined using GraphPad Prism analysis software with one-way ANOVA and Tukey’s multiple comparison test (* indicates significance compared to SEB+Vehicle).

We next tested if the decreased inflammation seen in the livers of SEB-immunized mice treated with I3C or DIM resulted from induction of apoptosis. In order to test this, we cultured splenocytes activated with SEB in the presence or absence of I3C and DIM. After 24 and 48 hours, we collected and counted the number of cells in each experimental group. At both time points, there was a significant decrease in the number of cells isolated from cultures treated with either I3C or DIM (Fig. 3A-B). Next we tested to see if I3C and DIM were inducing apoptosis in cells. Fig. 3C shows a representative experiment with cells labeled for Annexin V/PI, and Fig. 3D shows TUNEL staining while Fig. 3E shows DiOC6 staining, with data from 3 independent experiments compiled. Overall, these data together suggested that cells treated with SEB+I3C or DIM showed higher levels of apoptotic cells when compared to SEB+vehicle treated cells. The background apoptosis seen in cells treated with SEB+vehicle may result from T cells undergoing activation-induced cell death. Because DiOC6 staining detects cells undergoing apoptosis through mitochondrial pathway, our data also suggested that I3C and DIM may induce apoptosis in SEB-activated cells primarily through intrinsic pathway.

### Inhibition of caspase-2 blocks I3C or DIM-mediated apoptosis in SEB-activated cell, but does not affect the ability of these compounds to reduce activation and proinflammatory cytokine release

**Fig 4:**
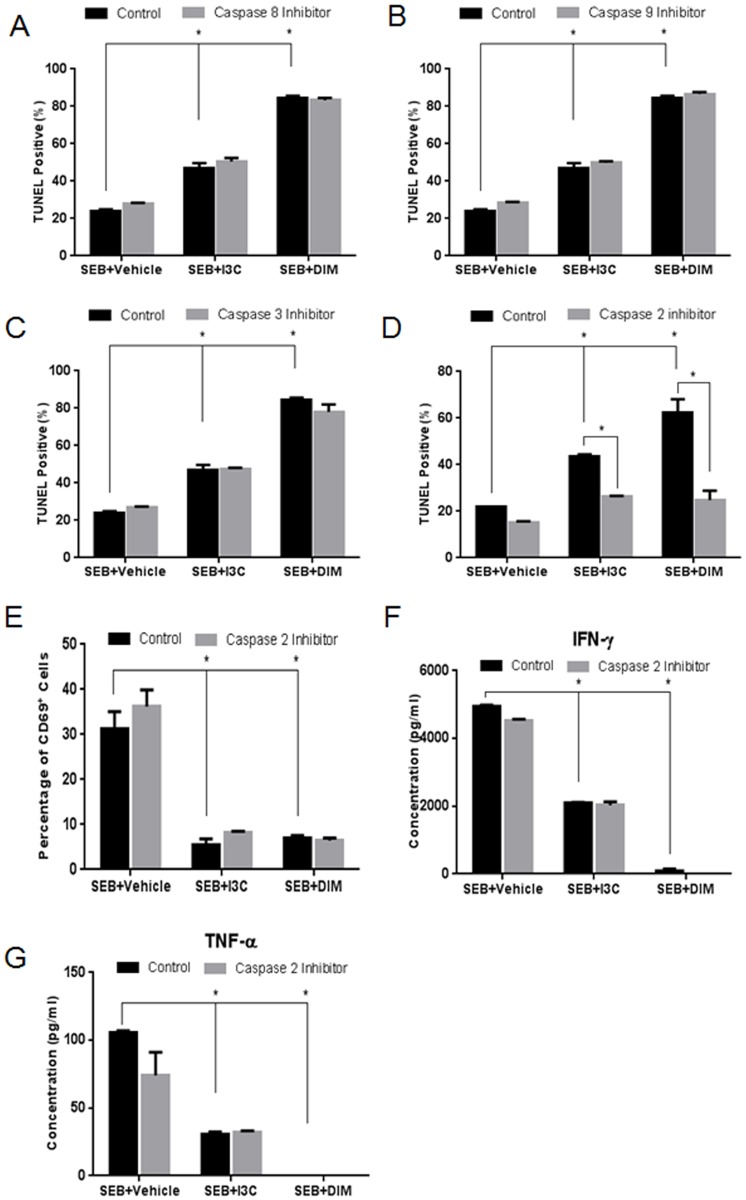
Role of caspase-2 in I3C and DIM-mediated apoptosis, cell activation, and cytokine release. Splenocytes from C57BL/6 mice were cultured and activated with 1μg/ml of SEB, in the absence or presence of I3C or DIM (100µM). The cultures were treated with 100µM of inhibitors for caspases-8 (A), -9 (B), -3 (C), or-2 (D). Percentage of TUNEL positive cells was determined using a TUNEL kit and analyzed by flow cytometry. Percentage of CD69 expressed in experimental groups was determined in the presence or absence of caspase-2 inhibitor (E). ELISAs were used to detect cytokine concentrations from supernatants collected from 24-hour cultured experimental groups treated with or without capase-2 inhibitor for IFN-γ (F) and TNF-α (G). Statistical significance was determined using GraphPad Prism analysis software with one-way ANOVA and Tukey’s multiple comparison to test between different groups. Two-way ANOVA and Sidak’s multiple comparison test were used to compare controls with those containing inhibitors. Significance is indicated by * and has a p-value <0.05.

In order to better understand the mechanism involving I3C and DIM-mediated apoptosis in cells stimulated with SEB, we performed a series of inhibitor studies *in vitro* and analyzed these cultures for apoptosis by TUNEL assay. Caspase-8 is known to play a key role in the initiation of the death cell receptor (extrinsic) pathway [28]. Our data using the caspase-8 inhibitor failed to show any change in the ability of I3C and DIM to induce apoptosis in SEB-activated cells when compared to controls without caspase-8 inhibition (Fig. 4A). Interestingly, inhibition of caspase-9, a prominent initiator of the mitochondrial pathway [29] and inhibition of caspase-3, an “executioner” caspase linked to both the intrinsic and extrinsic pathways [30], also failed to inhibit I3C and DIM-mediated apoptosis when compared to controls (Fig. 4B and C respectively). Lastly, we looked at inhibition of caspase-2, which is similar to caspase-9 in structure, and has been shown to induce cell death in response to pathogenic bacteria such as *S*. *aureus* [31]. Interestingly, inhibition of this particular caspase almost completely blocked I3C and DIM from inducing apoptosis in SEB-activated cells when compared to controls (Fig. 4D). This indicated that caspase-2 played a prominent role in I3C and DIM-mediated apoptosis in SEB-activated splenocytes.

In the current study, we noted that I3C and DIM reduced cytokine production as well as mediated caspase-2 dependent apoptosis in SEB-activated cells. We next determined if caspase-2 activation by either I3C or DIM played any role in T cell activation and cytokine production by SEB. In order to test this, we treated SEB-stimulated *in vitro* cultures of splenocytes with either I3C or DIM as described above, in the presence or absence of caspase-2 inhibitor. We first analyzed the activation marker CD69 by flow cytometry at 24 hours (Fig. 4E). Cells treated with I3C or DIM showed marked reduction in CD69 expression when compared to SEB+vehicle groups. In all these cultures, addition of caspase-2 inhibitor did not further alter the expression of CD69, thereby showing that I3C and DIM were down-regulating T cell activation independent of caspase-2. Next, we looked at production of IFN-γ and TNF-α, in supernatants from the aforementioned *in vitro* experiments (Fig. 4F-G). As with activation, I3C and DIM were still able to reduce these cytokines even in the presence of caspase-2 inhibitor. Together, these data indicated that caspase-2-inhibition does not affect the ability of I3C and DIM to reduce SEB-mediated T cell activation and inflammatory cytokine production.

### I3C and DIM increase activated caspase-2, and decrease bcl-2, in both *in vitro* and *in vivo* SEB models

**Fig 5:**
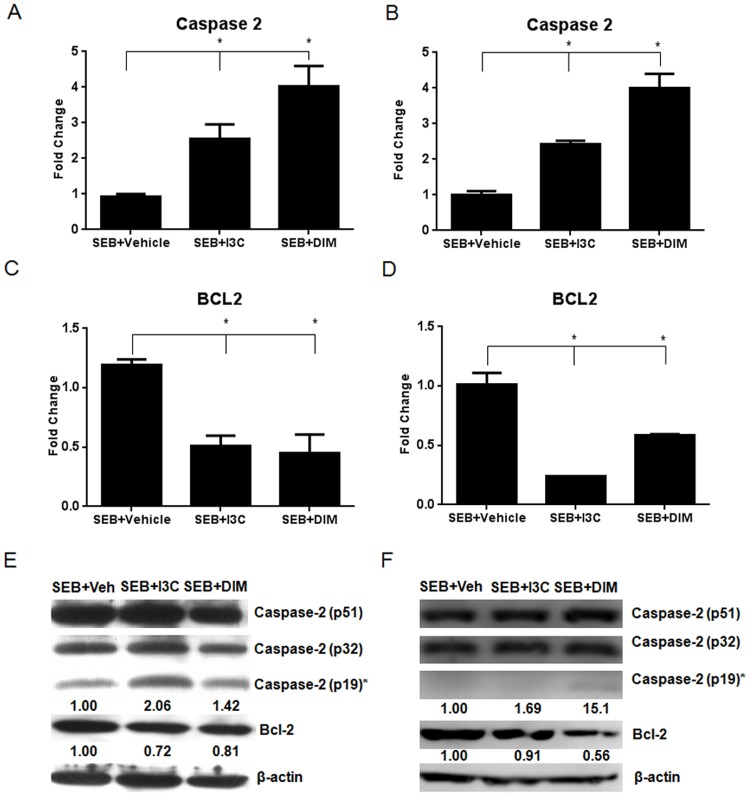
I3C and DIM increase the expression of caspase-2 and decrease Bcl-2 both in vitro and in vivo. Whole splenocytes from C57BL/6 mice were cultured and activated with 1μg/ml of SEB, in the absence or presence of I3C or DIM (100µM) for 12 hours. qRT-PCR was performed on RNA isolated from these *in vitro* cultures to determine expression of caspase-2 (A) and Bcl-2 (C). RNA was also isolated from *in vivo* isolated mononuclear cells collected from SEB-induced acute liver injury mice after 24 hours to determine expression of caspase-2 (B) and Bcl-2 (D). Protein expression of both caspase-2 and Bcl-2 was evaluated by western blotting in both *in vitro* (E) and *in vivo* (F) experiments. Relative expression of activated caspase-2 (p19) and Bcl-2 are shown below each respective blot under the appropriate bands. Numbers for relative expression were calculated by first normalizing expression levels to β-actin and comparing them to the vehicle group using ImageJ software. Statistical significance (p-value <0.05) was determined using GraphPad Prism analysis software with one-way ANOVA and Tukey’s multiple comparison test (* indicates significance compared to SEB+Vehicle).

Next, we studied the expression levels of caspase-2 both *in vitro* and *in vivo*. To this end, we isolated RNA from 12-hour *in vitro* cultures as described above and from *in vivo* mononuclear cells collected from our SEB-induced liver injury model. We performed qRT-PCR to look at the expression of caspase-2. In the *in vitro* treatments, there was a significant increase in the expression of caspase-2 in SEB+I3C or SEB+DIM groups when compared to SEB+vehicle group (Fig. 5A). We observed similar trend in the *in vivo* samples as well (Fig. 5B). Next, we studied the expression Bcl-2, an anti-apoptotic molecule. In both the *in vitro* and in vivo treated samples, we noticed a decrease in Bcl-2 expression in SEB+I3C or SEB+DIM groups when compared to SEB+vehicle group (Fig. 5C-D). These data suggested a reciprocal relationship between caspase-2 and Bcl-2 expression following I3C/DIM treatment.

To further corroborate these studies, we performed western blot under the same conditions as described above. For caspase-2, we focused on the expression of activated caspase-2, indicated by the cleaved p19 subunit as well as the intermediate cleaved p32 subunit. In *in vitro* samples, we saw increased expression of the cleaved product of caspase-2 (p19) in SEB+I3C and SEB+DIM groups when compared to SEB+vehicle group. Also, we noted that in these samples, Bcl-2 expression decreased in I3C/DIM-treated groups. Overall, similar observations were made using *in vivo*-exposed cells (Fig. 5F).

### I3C and DIM downregulate miRNA that are predicted to target caspase-2

**Fig 6:**
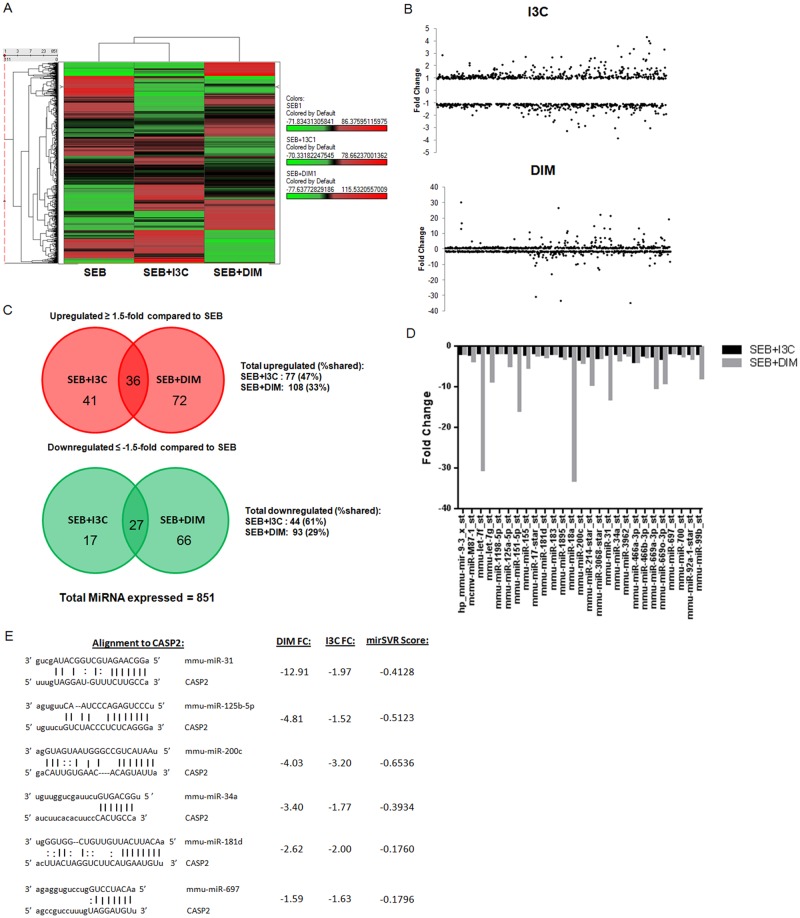
I3C and DIM downregulate potential miRNA that target caspase-2. Whole splenocytes from C57BL/6 mice were cultured and activated with 1μg/ml of SEB, in the absence or presence of 100µM of I3C or DIM for 12 hours, and miRNA was isolated from each group. Heatmap was generated compared SEB+Vehicle groups to either SEB+I3C or SEB+DIM (A). Dot plot was generated to depict fold change distributions of 851 detected miRNAs for groups treated with I3C (top) and DIM (bottom), indicating miRNAs that were up- and downregulated (B). Venn Diagrams were constructed to depict the number of up- (top) and downregulated (bottom) miRNAs that were ≥ 1.5-fold change in groups treated with I3C or DIM when compared to SEB+Vehicle (C). Bar graph depicting 27 miRNAs that both I3C and DIM downregulated with a ≥ 1.5-fold change (D). Table depicts the 6 miRNAs of the 27 shared between I3C and DIM that were predicted to target caspase-2 and had a miSVR of less than-0.1 (E). miSVR scores and alignment details were obtained from online miRNA database (microrna.org).

Because an increase in mRNA expression of caspase-2 in SEB-stimulated cells was observed after treatment with I3C/DIM, we examined the miRNA profile of samples from our *in vitro* cultures in hopes of identifying any miRNA that might be playing a role in this process. Total RNA was isolated from SEB+vehicle, SEB+I3C, and SEB+DIM cultures isolated after 12 hours, and relative expression of miRNA was determined using microarray miRNA analysis. A heatmap was constructed highlighting the differences in miRNA abundance between SEB+vehicle groups when compared to SEB+I3C and SEB+DIM groups (Fig. 6A). A dot-plot depicting the differential fold change expression of 851 miRNAs in groups treated with I3C/DIM compared to SEB+vehicle showed many miRNA in both treatment groups had a more than 1.5-fold change (Fig. 6B). Venn diagrams were constructed to highlight miRNAs that displayed at least a 1.5-fold change in treatment groups (Fig. 6C). Interestingly, around 50% or slightly more of the miRNAs dysregulated by I3C at least 1.5-fold change were shared with DIM. Samples treated with DIM had a more abundant and unique set of miRNAs that were significantly altered, only sharing around 30% 1.5-fold up- and downregulated miRNAs with I3C. We focused our attention on the 27 1.5-fold down-regulated miRNAs shared between I3C and DIM, which are depicted in a bar graph in Fig. 6D. Using an online miRNA target database (microrna.org), we searched if any of these 27 miRNAs that could target caspase-2. Among these potential miRNAs, we identified 6 candidates that had good alignment and were highly predicted to target caspase-2, as assessed by miSVR scores (Fig. 6E). These miRNA included miR-31, miR-125b-5p, miR-200c, miR-34a, miR-181d, and miR-697. These data suggested that I3C/DIM may induce caspase-2 by inhibiting miRNA that target, thereby promoting caspase-2-mediated apoptosis.

### I3C and DIM downregulate miR-31, which directly targets caspase-2 and affects apoptosis after SEB stimulation

**Fig 7:**
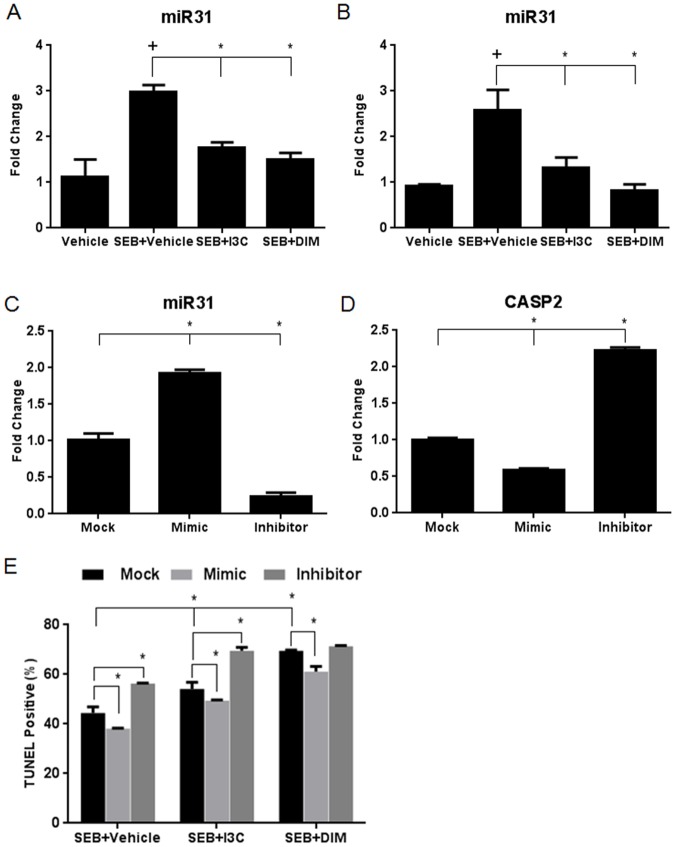
I3C and DIM downregulate the expression of miR-31, which targets caspase-2 and affects apoptosis in SEB-activated splenocytes. Total RNA was isolated from whole splenocytes cultured and activated with or without 1μg/ml of SEB, in the absence or presence of I3C or DIM (100µM) for 12 hours. RNA was also isolated from *in vivo* isolated mononuclear cells collected from SEB-induced acute liver injury mice after 24 hours. qRT-PCR was performed to determine expression miR-31 from *in vitro* (A) and *in vivo* samples (B). Transfections using miR-31 mimic and inhibitor were performed on *in vitro* splenocyte cultures activated with SEB. qRT-PCR was performed to validate successful transfection by examining miR-31 levels (C). Validation for miR-31 targeting of caspase-2 was performed by examining caspase-2 miRNA levels using qRT-PCR after transfection with mock (just transfection reagent), miR-31 mimic, or miR-31 inhibitor (D). TUNEL assay and flow cytometry were used to determine percentage of apoptosis in experimental groups compared to those containing mock, mimic, or inhibitor (F). Statistical significance (p-value <0.05) was determined using GraphPad Prism analysis software with one-way ANOVA and Tukey’s multiple comparison test (+ indicates significance compared to vehicle and * indicates significance compared to SEB+vehicle). Two-way ANOVA and Sidak’s multiple comparison test were used to compare experimental and control groups with those containing mock, mimic, or inhibitor. Significance is indicated by * and has a p-value <0.05.

We next focused our attention on how downregulation of miR-31 may play a role in I3C/DIM-induced up-regulation of caspase-2 because it was the most highly downregulated by DIM as assessed by microarray analysis, and miR-31 has been reported to play a role in apoptosis [32–33]. First, we validated our microarray data by examining miR-31 expression levels by qRT-PCR in our *in vitro* and *in vivo* experiments. We observed that miR-31 levels increased after exposure to SEB, however, both indole compounds were able to reduce these levels in *in vitro* cultures (Fig. 7A). This same trend was observed using the *in vivo* samples as well (Fig. 7B). After validating our microarray data, we next performed transfections in SEB-activated *in vitro* cultures using mimic and inhibitors for miR-31 to determine if this miRNA directly targeted caspase-2. The success of the transfection was observed when cultures given mimic displayed increased levels of miR-31 when compared to mock, and cultures given inhibitor resulted in decreased expression of this miRNA (Fig. 7C). With the success of the transfection confirmed, we next determined how caspase-2 mRNA levels changed under these conditions. Cultures that were given mimic showed a significant decrease in caspase-2 expression, and those given inhibitor displayed increased expression (Fig. 7D). These data confirmed that miR-31 targets caspase-2 levels, thereby explaining how I3C and DIM are able to upregulate caspase-2, by inhibiting the expression of miR-31.

Lastly, we wanted to determine the effect modulating miR-31 levels on apoptosis in our experimental groups. In order to address this, we cultured cells with SEB+Vehicle, SEB+I3C, or SEB+DIM in the presence or absence of either mock, mimic, or inhibitor, and examined apoptosis levels using the TUNEL assay (Fig. 7E). When compared to mock controls, we were able to show that transfection with the miR-31 mimic resulted in decreased levels of apoptosis in all experimental groups. When cultures were transfected with miR-31 inhibitor, there was a significant increase in apoptosis in SEB+Vehicle and SEB+I3C groups. While there was a slight increase in apoptosis in SEB+DIM groups transfected with inhibitor, this was not found to be significant. It is possible that DIM alone is able to decrease miR-31 levels substantially and transfecting these cultures with miR-31 inhibitor does not significantly modulate the levels of this miR to the point that apoptosis is altered. However, taken together, these data demonstrate that the modulation of miR-31 levels in cells stimulated with SEB can affect their ability to undergo apoptosis.

## Discussion

There is growing evidence that natural indoles, I3C and DIM, possess anti-inflammatory properties, making them promising candidates against toxicity induced by bacterial toxins, such as SEB. For example, recently DIM was shown to suppress lipopolysaccharide (LPS)-induced brain inflammation in the hippocampus of mice [34]. I3C was found to reduce cellular infiltration into the broncho-alveolar lavage fluid (BALF) of LPS-induced acute lung injury, while also decreasing proinflammatory cytokines, such as IL-6 and TNF-α, in the lungs [35]. Our laboratory has recently shown that I3C and DIM can suppress T cell activation by SEB through epigenetic regulation involving HDAC expression [21]. Moreover, these natural indoles were also found to suppress experimental autoimmune encephalomyelitis [18]. In the present study, we were able to further investigate the properties of these natural compounds against SEB-induced acute liver injury. Since C57BL/6 mice are more resistant to SEB [9], we sensitized these mice with Dgal to more closely resemble human exposure. Our data demonstrated that these indoles can reduce cellular infiltrating into the liver and decrease the production of proinflammatory cytokines IFN-γ, TNF-α, IL-6, and IL-2. To better understand how I3C and DIM were so effective at reducing inflammation caused by SEB, we also explored the ability of these compounds to induce apoptosis in SEB-activated cells.

Several studies have shown that I3C and DIM are capable of inducing apoptosis in a variety of cancer cells. Both I3C and DIM were shown to induce apoptosis in the human breast

cancer cell line MCF-10A by inhibiting the activation of Akt which reduced the binding of NF-κB [36–37]. I3C was also found to induce apoptosis in fungal *Candida albicans*, an opportunistic pathogen to humans, by the generation of reactive oxygen species, cytochrome *c* release, and activation of metacaspase [38]. In colon cancer cells, DIM was shown to activate caspases-3, -7, -8, and-9, increase translocation of cytochrome *c*, while also reducing the anti-apoptotic Bcl-2 protein [39]. Most of these studies indicated that I3C and DIM mediate apoptosis primarily involving the mitochondrial pathway.

In the present study we also experimentally showed through DiOC6 staining that I3C and DIM induce apoptosis in SEB-activated cells via the intrinsic pathway. Surprisingly though, our inhibitor studies failed to indicate that caspases-3, -8, or-9 played significant roles in this process. However, inhibition of caspase-2 greatly reduced the ability of these compounds to induce apoptosis in SEB-activated splenocytes. We also showed reciprocal expression between caspase-2 and Bcl-2 in our experiments, which is important inasmuch as Bcl-2 has been shown to block caspase-2-mediated apoptosis [40]. In our previous study, we noted that I3C and DIM act as class I HDAC inhibitors [21], and furthermore, inhibition of these class-specific HDACs has been reported to lead to increased caspase-2 activity and mitochondrial cell death [41]. Nonetheless, the current report shows for the first time that caspase-2 may play a critical role in I3C and DIM-mediated apoptosis.

Despite being one of the most evolutionarily conserved caspases, caspase-2 function remains controversial and poorly understood. Whether or not caspase-2 fits the role of a traditional initiator or effector caspase, or both, is still unknown. However, this caspase has been shown to regulate and be induced in a variety of cellular death processes, such as endoplasmic reticulum (ER) stress, DNA damage, and metabolic imbalance [31]. It has been suggested that caspase-2 induced apoptosis is dependent on the engagement of the mitochondrial pathway since this caspase does not directly process or activate executioner caspases. Caspase-2 can then release cytochrome c to activate the Apaf-caspase-9 apoptosome to initiate apoptosis [42]. However, in our studies, caspase-9 inhibition failed to affect I3C and DIM-mediated apoptosis, indicating that the caspase-2-mitochondrial dependent apoptosis induced by these compounds could be independent of cytochrome *c* or further caspases. It has been shown that caspase-2 can engage the mitochondrial pathway to induce apoptosis via the caspase-independent death effectors apoptosis-inducing factor (AIF) and endonuclease G [43]. Therefore, this is a possible way in which I3C and DIM engage caspase-2 in the induction of the mitochondrial pathway, independent of caspase-9. Because caspase-2 is also the sole caspase known to translocate from the cytosol to the nucleus, it has been postulated that this caspase is involved in cellular processes other than apoptosis [44]. In our studies, we did not find a role for caspase-2 in mediating I3C or DIM-mediated decrease in SEB-induced immune cell activation or proflammatory cytokine release.

It is particularly interesting to observe the role miRNAs might be playing in the process highlighted in the current study, particularly that of miR-31. The influence of miRNAs in a variety of mechanisms has begun to emerge since its discovery a little more than a decade ago. These noncoding endogenous RNA molecules have been shown to play a major role in the immune response [45], including autoimmunity and inflammation [46]. Recently, our lab published data showing how miR-155 was able to promote lung inflammation after exposure to SEB by targeting suppressor of cytokine signaling 1 (SOCs1) [47]. In the current study, we noted that the indoles altered the expression of several miRs. Of these, we were able to characterize miR-31 to play a critical role because treatment with indoles led to significant down-regulation of miR-31 and consequent induction of caspase-2. Using transfection experiments with mimic or inhibitors, we were able to confirm that miR-31 did selectively target caspase-2, and the modulation of miR-31 levels affected apoptosis in splenocytes that were activated with SEB. While our report linking miR-31 to caspase-2 appears to be the first, miR-31 has already been shown to influence apoptosis, although there are conflicting reports as to whether this miRNA promotes or inhibits this particular process. In prostate cancer cells, the downregulation of miR-31 was found to promote resistance to chemotherapy-induced apoptosis [48]. Alternatively, in human breast cancer cells, miR-31 was shown to sensitize these cells to apoptosis by directly targeting protein kinase C epsilon [32]. In non-small cell lung cancer, miR-31 was also shown to exert anti-apoptotic properties by inhibiting ABCB9, a gene known to play a role in drug resistance [33].

SEB remains to be a significant threat, particularly to humans inasmuch as even low doses can cause lethal toxic shock [5]. This toxin is a potent simulator of the immune system and since the bacteria that produce these toxins continues to show impressive resistance to antibiotics, it is important to discover new treatments aimed at suppressing the inflammatory responses initiated by SEB exposure. As with our previous report [21], the current study shows that I3C and DIM, naturally-occurring indole compounds found in cruciferous vegetables, are both effective at reducing activation of immune cells and the cytokine storm produced by SEB.

In our previous study, we noted that inhibitors of histone deacetylase class I (HDAC-I), but not class II (HDAC-II), showed significant decrease in SEB-induced T cell activation and cytokine production [21]. In addition, I3C and DIM caused a decrease in HDAC-I but not HDAC-II in SEB-activated T cells, thereby suggesting that I3C and DIM may inhibit SEB-mediated T cell activation by acting as HDAC-I inhibitors. These data suggested that I3C and DIM trigger epigenetic modulations to suppress SEB-induced inflammation. In the current study, we also noted that I3C and DIM can activate caspase-2 and consequent apoptosis. Moreover, we identified an interesting role miR-31 seemed to play in cells activated with SEB. Because caspase-2 inhibitor failed to suppress T cell activation and cytokine production, our studies suggest that I3C and DIM may use multiple pathways to suppress inflammation. Thus, our studies suggest for the first time that plant-derived indoles are potent suppressors of SEB-induced T cell activation and cytokine storm and that they may mediate these effects by acting as HDAC inhibitors as well as inducing apoptosis in activated T cells through activation of caspase-2, assisted in part by their modulation of miRs. This may account for the dramatic decrease in inflammatory cells seen in the liver of mice exposed to SEB after being treated with I3C or DIM.

## Supporting Information

S1 Fig Chemical structures of I3C and DIM. (TIF)
