# Supplementary figures and images for: Natural Indoles, Indole-3-Carbinol (I3C) and 3,3’-Diindolylmethane (DIM), Attenuate Staphylococcal Enterotoxin B-Mediated Liver Injury by Downregulating miR-31 Expression and Promoting Caspase-2-Mediated Apoptosis
Source: PLoS One. 2015 Feb 23;10(2):e0118506. doi: 10.1371/journal.pone.0118506 (PMC4338211; doi:10.1371/journal.pone.0118506)

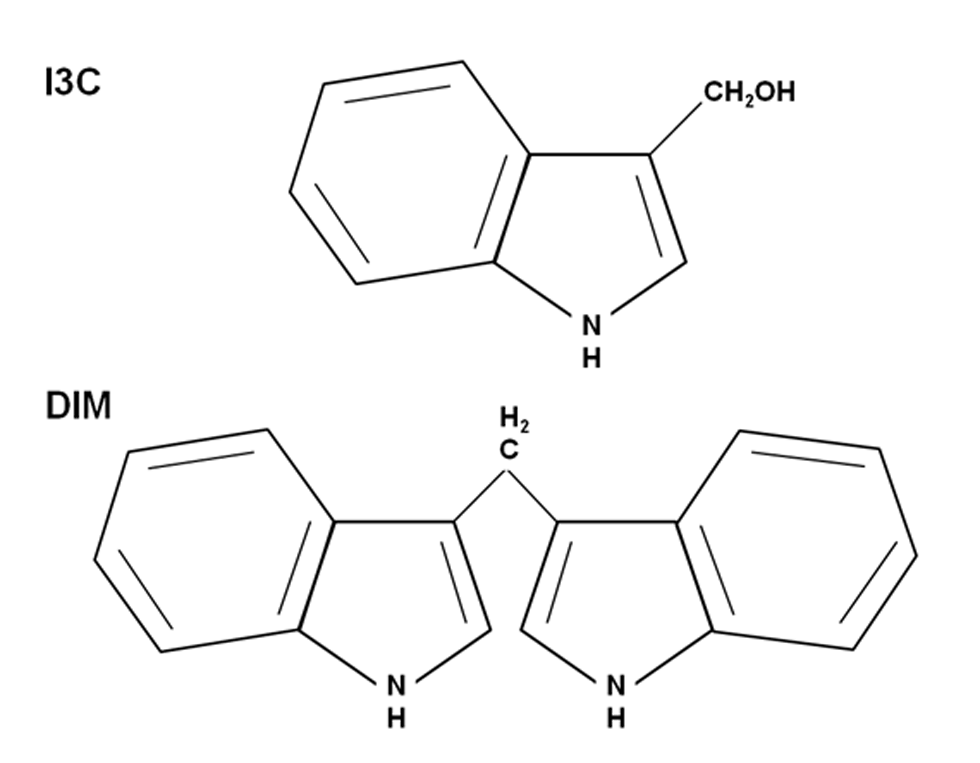

Supplement: S1 Fig — (TIF) [file pone.0118506.s001.tif]
